# Theoretical and Experimental Research on Multi-Layer Vessel-like Structure Printing Based on 3D Bio-Printing Technology

**DOI:** 10.3390/mi12121517

**Published:** 2021-12-06

**Authors:** Huanbao Liu, Xianhai Yang, Xiang Cheng, Guangxi Zhao, Guangming Zheng, Xuewei Li, Ruichun Dong

**Affiliations:** College of Mechanical Engineering, Shandong University of Technology, Zibo 255000, China; chengxiang@sdut.edu.cn (X.C.); zgx2019@sdut.edu.cn (G.Z.); zhengguangming@sdut.edu.cn (G.Z.); lixuewei@sdut.edu.cn (X.L.); Dongruichun@sdut.edu.cn (R.D.)

**Keywords:** vessel-like, 3D bioprinting, synchronous relationship, multi-material

## Abstract

Cardiovascular disease is the leading cause of death worldwide. Traditional autologous transplantation has become a severe issue due to insufficient donors. Artificial blood vessel is an effective method for the treatment of major vascular diseases, such as heart and peripheral blood vessel diseases. However, the traditional single-material printing technology has been unable to meet the users’ demand for product functional complexity, which is not only reflected in the field of industrial manufacturing, but also in the field of functional vessel-like structure regeneration. In order to achieve the printing and forming of multi-layer vessel-like structures, this paper carries out theoretical and experimental research on the printing and forming of a multi-layer vessel-like structure based on multi-material 3D bioprinting technology. Firstly, theoretical analysis has been explored to research the relationship among the different parameters in the process of vessel forming, and further confirm the synchronous relationship among the extrusion rate of material, the tangential speed of the rotating rod, and the movement speed of the platform. Secondly, sodium alginate and gelatin have been used as the experimental materials to manufacture the vessel-like structure, and the corrected parameter of the theoretical analysis is further verified. Finally, the cell-loaded materials have been printed and analyzed, and cell viability is more than 90%, which provides support for the research of multi-layer vessel-like structure printing.

## 1. Introduction

According to relevant reports [1], the organ donation rate in China has reached 2/million, and the shortage of organ transplant donors is serious. With the development of 3D bio-printing technology, in vitro tissue or organ reconstruction has become an effective means to solve the shortage of donors, and is also an important direction in the field of tissue engineering [2]. In recent years, important achievements have been made in the regeneration of blood vessels, cartilage, heart, and other organs. Angiogenesis [3] or manufacturing technology [4,5,6] is an important means of interventional therapy for vascular diseases, which has a broad application prospect in the treatment of vascular diseases due to its advantages of biological adaptability and immunity. Due to the diversity of vascular structures, traditional single-material printing technology [7] has been unable to satisfy the demand of vessel-like printing. Therefore, an effective printing method is urgently needed to realize the forming and manufacturing of multi-layer vessel-like structures [8].

The relevant research results show that controlling biomaterials or cells to be distributed and printed on demand with sufficient precision is the key point to achieve high-precision vessel-like structure regeneration manufacturing [9]. The traditional vessel-like structure manufacturing and forming technology usually inoculates cells on the surface of biomaterials to achieve the formation of a vessel-like structure. The forming process is relatively simple, but due to the influence of human factors or manual manipulation, the precision of inoculation is not easy to control [10]. Therefore, there is an urgent need for effective angioplasty methods to achieve the regeneration of the vascular structure.

At present, the main methods used for vessel-like structure formation are the mold method and the 3D bioprinting method. The mold method involves adopting the mold obtained by assembling fixed parts and realizing the formation of a vessel-like structure by adding biological materials into the cavity. The mold-forming method has been adopted by Lee et al. [11] to achieve the effective forming of the cell microsphere. Then, Xu et al. [12] used the mold-forming method to fabricate a multi-layer vessel-like structure. In order to achieve the printing and molding of vessel structures with higher accuracy, 3D printing molding with the function of on-demand printing has been widely applied in the fields of tissue engineering and organ regeneration. Delrot et al. [13] used inkjet 3D bioprinting to achieve the forming mechanism of microspheres and the distribution of cell microspheres on demand, and Tan et al. [14] have employed this approach to manufacture a micro-vessel-like structure. Laser-assisted 3D bioprinting was used by Xiong et al. [15], which has realized the formation of vessel-like structures. Coaxial focused wrapping technology has also been applied in the 3D printing of extrusion molding for the forming and manufacturing of vascular structures. The extrusion molding 3D printing methods all adopt coaxial focusing printing methods, such as “dual-material synchronous extrusion” [16,17] and “micro-channel extrusion” [18,19], to realize the printing and molding of the vessel structure. However, because the density and uniformity of cells in the vessel-like structure are mainly determined by the density and uniformity of cells in the biological materials, it is not easy to meet the requirements of cell microsphere shaping and printing-on-demand.

In summary, 3D bioprinting technology is an effective method for vascular structure manufacturing, and on-demand printing technology is a key technology for high-precision cell microsphere distribution and vessel-like molding. However, the existing 3D bio-printing technology with the function of on-demand printing has seldom been studied on the forming process of vascular structures under the action of multi-parameter coupling, and even less so on the on-demand printing process of cell microspheres in extrusion 3D printing, which has affected the distribution accuracy of cell microspheres and the regeneration of high-precision vessel-like structures.

## 2. Theory, Fabrication Methods, and Materials

### 2.1. Theory

**Figure 1:**
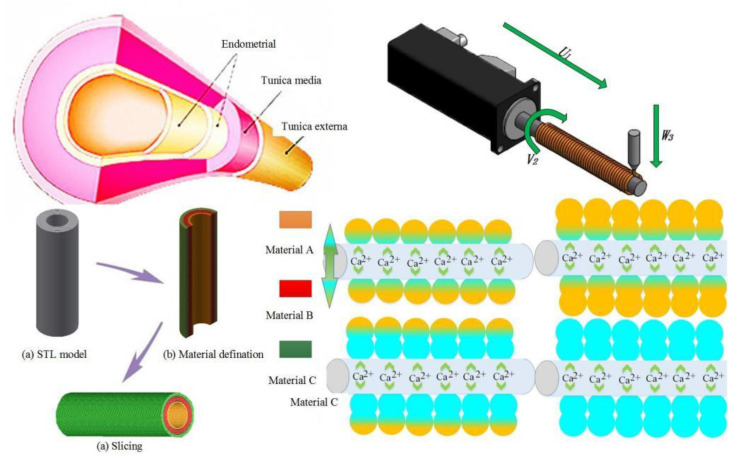
Vascular structure and multi-material vascular structure forming process.

Blood vessels are composed of multiple layers of different cells, as shown in Figure 1. Different layers and different cells need to be distributed and printed to form a similar vessel-like structure. However, theoretical research on the formation of single-layer vessel-like structures has certain limitations, so it is necessary to carry out theoretical and experimental research on the printing of multi-layer vessel-like structures.

Based on the preliminary analysis and experimental research on the theoretical research of single-layer printing synchronization [20], in order to carry out the printing molding of a multi-layer vessel-like structure, this paper conducted further research on multi-material printing in vessel-like molding through theoretical analysis. As shown in Figure 1, the multi-layer vessel-like structure is divided into endometrial, tunica media, and tunica extema layers. In order to successfully print vessel-like structures, three different types of cells are extruded to achieve the construction of vessel-like structures.

The outer diameter of the single-layer vessel-like structure is considered as:

(1) $$\begin{array}{c} H=\alpha \left(\sqrt{{\left(WL/2U\right)}^{2}-L^{2}}-R\right)\\ H=\alpha \left(\sqrt{{\left(WC/2V\right)}^{2}-L^{2}}-R\right) \end{array}\}$$

where *U* is the moving speed of the nozzle, *V* is the rotating speed of the motor, *W* is the extrusion speed of the material, and *R* is the diameter of the rotating rod.

**Figure 2:**
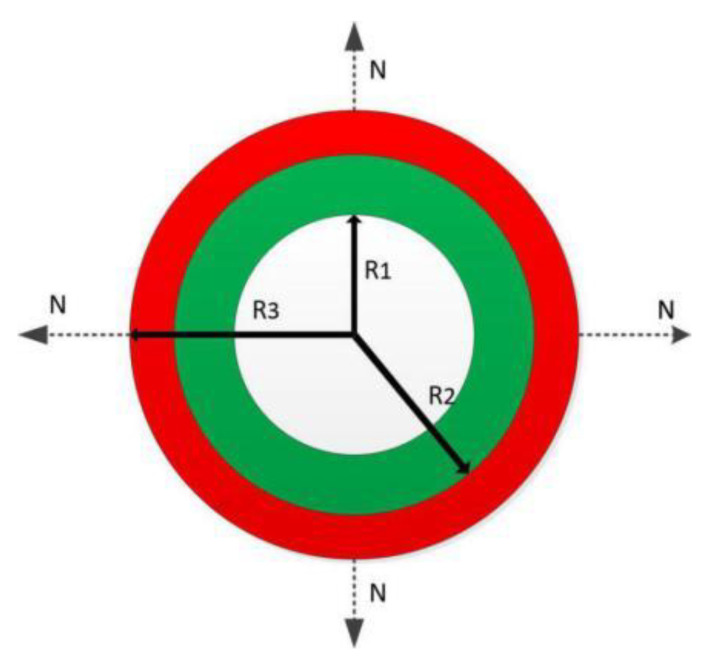
Schematic diagram of multi-layer vessel-like structure construction.

When the number of printing layers exceeds two layers (≥2), as shown in Figure 2, the theoretical formula is:

(2) $$\begin{array}{c} H_{n}=\sum_{n=1}\alpha_{n}\left(\sqrt{{\left(W_{n}L/2U_{n}\right)}^{2}-L^{2}}-R_{n}\right)\\ H_{n}=\sum_{n=1}\alpha_{n}\left(\sqrt{{\left(W_{n}C/2V_{n}\right)}^{2}-L^{2}}-R_{n}\right) \end{array}\}(n=1,2,3,4\ldots )$$

where *U_n_* is the movement speed of the nozzle, *V_n_* is the rotation speed of the motor, *W_n_* is the extrusion speed of the material, and *R_n_* is the sum of the rotating rod diameter and the single-layer vessel-like structure thickness when the number of printing layers exceeds two layers (≥2).

The control of film thickness has been treated as an important parameter to evaluate the molding quality of the vessel-like structure. The film thickness of each layer should be kept constant ($R_{n}-R_{n-1}=\Delta h$) under the ideal vessel-like structure test. When printing the second vessel-like structure, the extrusion speed of the material and the tangential speed of the rotating rod should be controlled to remain unchanged ($W_{1}=W_{1}^{\prime }$, $V=V^{\prime }$). Based on $v=wR_{1}$, if the extrusion speed of the material and the rotation tangential speed of the rotating rod remain unchanged, the rotation speed of the rotating rod should be controlled. It can be seen that the rotation speed of the rotating rod decreases with the increase of the number of printing layers, as follows:

(3) $$w_{n}^{\prime }=\frac{w_{1}R_{1}}{R_{1}+(n-1)*\Delta h}\;n=1,2,3\ldots$$

The moving speed of the nozzle decreases with the increase of the outer diameter, as shown in Equation (4):

(4) $$U_{n}=\frac{w_{1}*R_{1}*R}{\pi [R_{1}+(n-1)*\Delta h]}\;n=1,2,3\ldots$$

where *R*_1_ is the radius of the rotating rod, *R* is the radius of the nozzle, Δh is the layer thickness of the printing layer, and $U_{n}$ is the moving speed of the nozzle after nth layers of printing.

### 2.2. Fabrication Methods

**Figure 3:**
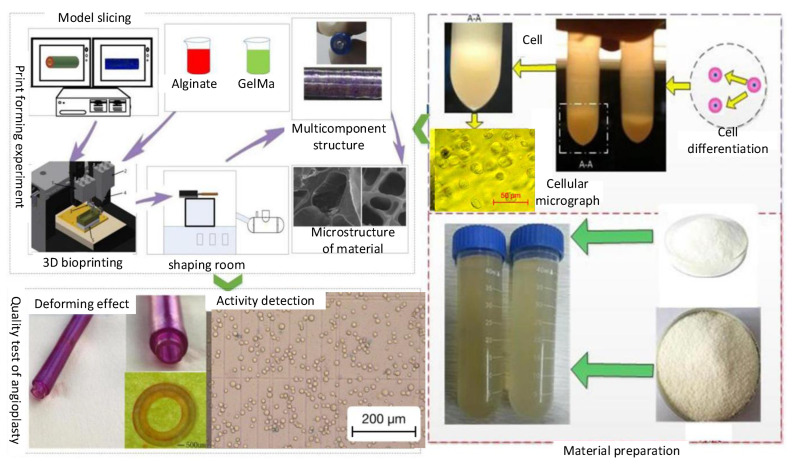
Multi-nozzle 3D bioprinting process.

**Figure 4:**
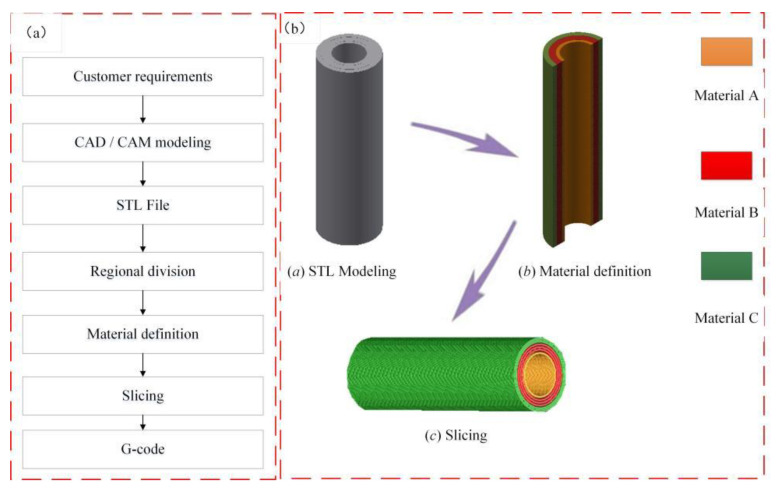
Vascular structure construction. (**a**) Model building process; (**b**) Material distribution in different areas of the vessel-like structure.

In the process of constructing a vessel-like structure with 3D biological vessel-like printing technology (Figure 3), the different layers’ definition of the blood vessel is of great significance to the construction of the vessel-like structure. The construction process of the vessel-like structure model is shown in Figure 4a. According to the relevant requirements of customers, CAD/CAM software was used for entity modeling, and then the STL file of the 3D CAD model was obtained according to the actual vessel-like or tissue structure model. Secondly, the three-dimensional model is divided into different regions, and then the different areas are processed with different materials by slicing software. The inner layer is defined as material A, the middle layer is material B, and the outer layer is material C, as shown in Figure 4b. Finally, the vessel-like structure was sliced to form the corresponding G code. The vessel-like model designed by CAD/CAM has been formed by stacking and distributing the materials orderly, with multi-material 3D vessel-like bioprinting.

### 2.3. Materials

In order to construct the multi-layer vessel-like structure, sodium alginate (purchased from Longood Medicine Co., Ltd., Beijing, China) and gelatin materials (purchased from Longood Medicine Co., Ltd., Beijing, China), which are natural materials, harmless to cells, were used as experimental materials. The vessel-like structure printing test was carried out with sodium alginate concentrations of 1, 1.5, 2, 2.5, 3, 3.5, and 4 wt.%, and gelatin concentrations of 2, 4, 6, 8, and 10 wt.%. The diameters of the rotary rod selected in the test were 2, 2.5, 3, 3.5, 4, 4.5, 5, 5.5, 6, 6.5, and 7 mm. The vascular structure was constructed at 4 °C. Gelatin is a temperature-sensitive gel, which can greatly improve the stability of vascular structure when mixed with sodium alginate. At the end of printing, the mixed materials (mass ratio 1:1) are chemically crosslinked with calcium ions.

## 3. Results

### 3.1. Multi-Layer Printing Path Planning

Traditional 3D printing technology has adopted the stacking printing molding method to manufacture vessel-like structures, which has the characteristics of a wide printing space without hindrance. However, the new multi-material vessel-like 3D bioprinting has a perfect rotating printing molding area, the printing molding range is relatively small, and the printing process is hindered. Therefore, it is necessary to plan the printing path so as to overcome the obstacles in the printing process and realize the accurate positioning of the needle position under the minimum moving path, which has laid a foundation for the printing of vessel-like structures.

**Figure 5:**
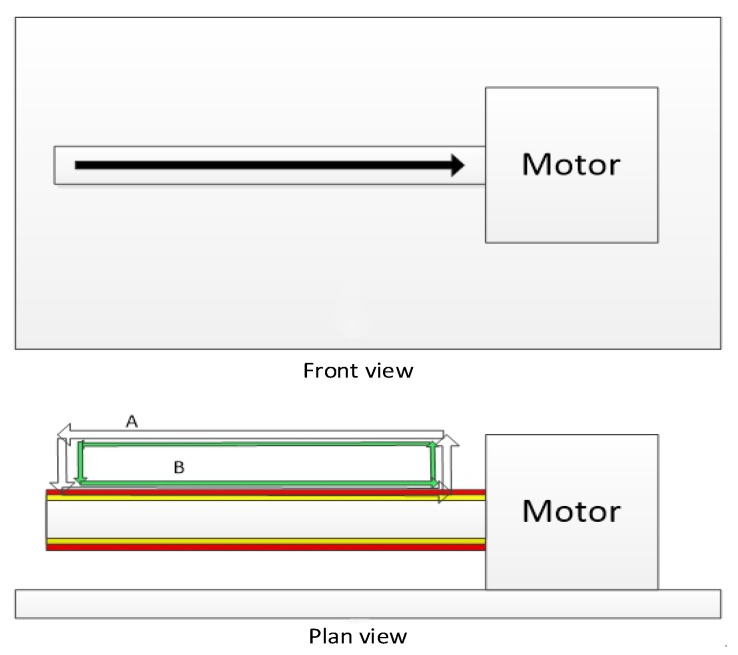
Print path planning of the nozzle. (**A**) Nozzle A movement path and (**B**) nozzle B movement path.

Before the printing process begins, all the motion axes should be returned to zero, the absolute spatial position of the rotating rod should be determined, the corresponding G code should be generated, and the generated G code can be written. Based on the requirement of multi-layer printing, after the printing of the first layer is fabricated, the height of the Z-axis (equal to the extrusion diameter of the material) should be raised according to the actual printing requirements, and then the printing experiment of the second layer should be carried out. Under the condition of keeping the output diameter of the sprinkler head unchanged, during the printing process, every layer of the printhead is increased and kept unchanged, thus realizing the formation of a double-layer vascular structure, as shown in Figure 5.

### 3.2. Multi-Layer Vessel-like Structure Printing Experiment

**Figure 6:**
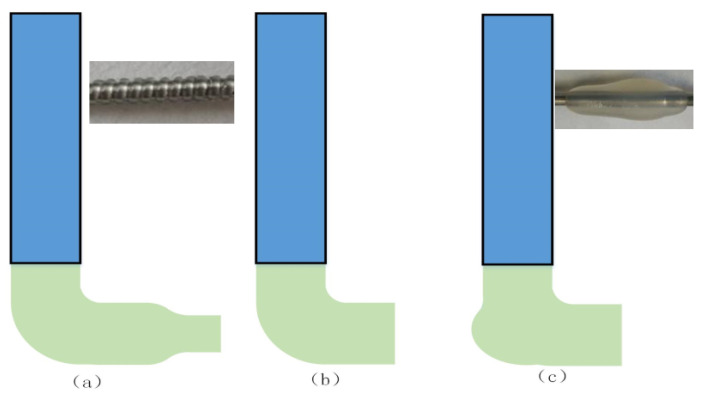
The initial shape of the extruded material. (**a**) *W > U*, (**b**) *W ≈ U*, (**c**) *W < U.*

In the process of the multi-layer vascular structure printing molding experiment, the control relationship of the parameters will affect the extrusion morphology of the material, as shown in Figure 6. When the extrusion speed of the material is less than the moving speed of the nozzle, the material will show an obvious “stretching” phenomenon and the forming diameter of the material will be less than the extrusion diameter (Figure 6a). When the extrusion speed of the material is matched with the moving speed of the nozzle, the forming diameter of the material will be approximately equal to the extrusion diameter (Figure 6b). When the extrusion speed of the material is greater than the moving speed of the nozzle, the material will show an obvious “heel” phenomenon (Figure 6c), which will affect the extrusion accuracy of the material. Therefore, the phenomena of “stretching” and “heel” should be avoided in the experiment.

The construction of the vascular structure is a relatively complicated process, which includes many key forming parameters, such as multi-parameter coordinated control, temperature maintenance, and environmental factors. In order to realize the multi-parameter coordinated control, we have analyzed the multi-parameter theoretical relationships.

**Figure 7:**
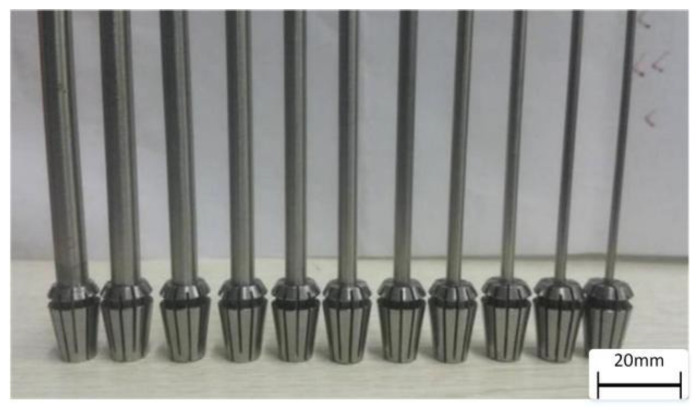
The different diameters of the rotating rods in the experiment.

**Figure 8:**
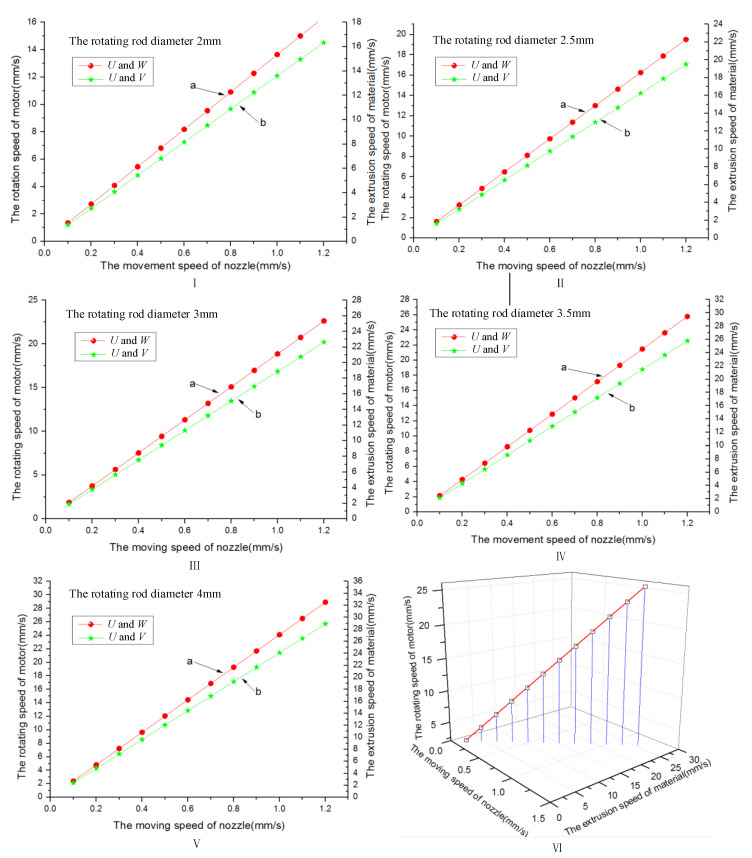
(**I**) The rotating rod diameter 2 mm. (**II**) The rotating rod diameter 2.5 mm. (**III**) The rotating rod diameter 3 mm. (**IV**) The rotating rod diameter 3.5 mm. (**V**) The rotating rod diameter 4 mm. (**VI**) The relationship among motor rotating speed, nozzle moving speed and material extrusion speed under the different rotating rod diameter.

In the experiment, we adopted different diameters of the rotation rod to construct the vessel-like structure (Figure 7). Figure 8 shows the relationship between the nozzle extrusion speed, the rotating speed of the rotary motor, and the motion speed of the linear motor. With the increase of the nozzle velocity, *U*, the rotational speed, *V*, of the rotating motor and the extrusion speed, *W*, of the material both show a significant increasing trend; therefore, there is a fixed matching relationship between different printing parameters.

**Figure 9:**
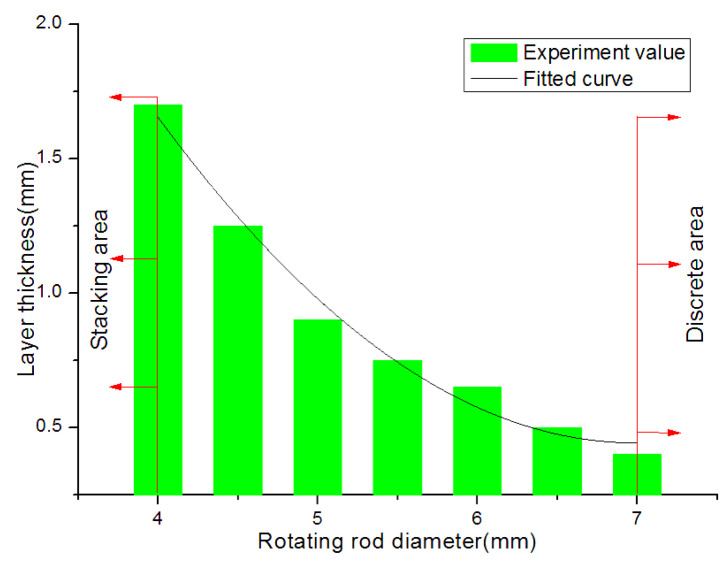
The relationship between film thickness and rotating rod diameter.

Based on the above theoretical analysis results, we have used 6% gelatin and 3% sodium alginate as experimental material to observe the relationship among linear motor speed, motor rotation speed, and material extrusion speed. When the rotation of the rod diameter is 7 mm, the rotating rod optional speed is 1.4 r/s, the nozzle move speed is 1 mm/s, and the material extrusion speed is 5 mm/s. The layer thickness has been analyzed under the different rotation rod diameters, and the analysis results are shown in Figure 9. It can be seen that the film thickness decreases with the increase of the diameter of the rotating rod. When the rotating rod diameter is less than 4 mm, the material extrusion velocity is much greater than the tangential velocity of the rotating rod, which has easily caused the phenomenon of vessel-like structure accumulation. When the rotating rod diameter exceeds 7 mm, the extrusion material will be separated, and the vessel-like structure will not be formed. According to the formula v=wR, the reason why the vessel-like structure cannot be formed is that the tangential velocity of the rotating rod increases with the increase of the diameter of the rotating rod under the condition that the rotating motor speed w remains unchanged; thus, the vessel-like structure cannot be formed.

**Figure 10:**
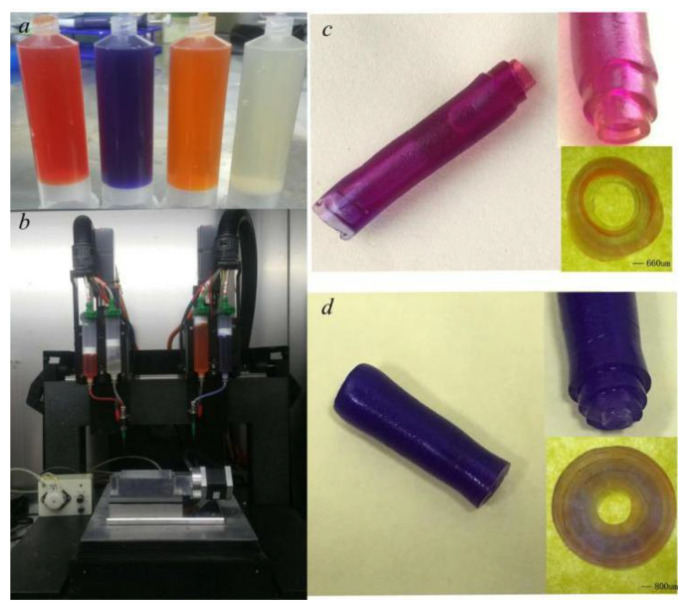
Vessel-like structure printing experiment: (**a**) materials, (**b**) 3D bioprinter, (**c**) vessel-like structure with three layers, and (**d**) vessel-like structure with four layers.

Based on the above analysis results, the multi-material 3D vessel-like bioprinting has been used to fabricate the vessel-like structure, as shown in Figure 10. When the nozzle moving speed and motor rotating speed are 1 mm/s and 1.4 r/s respectively, the deforming effect of the three-layer vessel-like structure is as shown in Figure 10c, where it can be seen that the different layers have different thicknesses. When the nozzle moving speed and motor rotating speed are 1 mm/s and 1.4 r/s respectively, the material extrusion speed increases with the external diameter of the vessel-like structure, and the different layers have similar layer thicknesses, as seen in Figure 10d. It can be seen that keeping the synchronization relationship can control the consistency of layer thickness.

**Figure 11:**
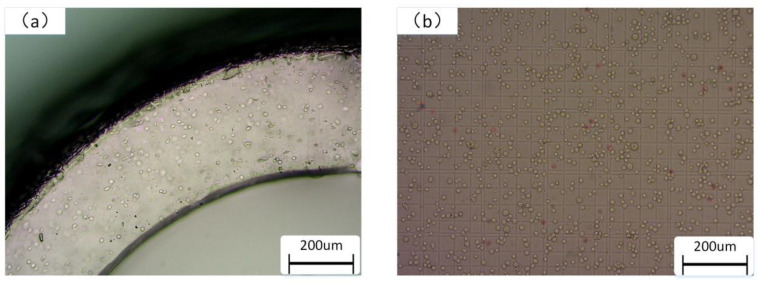
Cell survival of cell-laden vessel-like structure. (**a**) The printed cells distributed in the vascular structure and (**b**) cell survival rate statistics.

According to the operation flow of cell printing, the single-layer vascular structure printing test was carried out on the biomaterial-containing cells. Firstly, the mixed material of 2% sodium alginate and 6% gelatin was prepared as the test material, and the material was filtered to remove impurities and bacteria. Secondly, the renal epithelial cell line (293) was selected as the cell material for the vascular structure printing test. The number of differentiated cell particles was mixed with 2% sodium alginate and 6% gelatin in proportion to make the concentration of cell particles reach 1 × 10^7^/mL. Then, the cell printing test was carried out. During the printing process, the printing environment was controlled to prevent the phenomenon of biomaterial blocking the nozzle. The forming effect of the printed vascular vessel-like structure was relatively good, and the surface uniformity was high. The cells in the printed vessel-like structure were observed through an optical microscope (primotech), as shown in Figure 11. The printed cells were evenly distributed in the vascular structure. The vessel-like structure has been degraded by citric acid, and then the cells have been stained with Trypan blue, whereby the dead cells will turn blue (Figure 11b), and the cell survival rate was statistically analyzed by Image Pro Plus image analysis software. We can draw a conclusion that the printed cells’ survival rate can reach 90%.

## 4. Conclusions

In this paper, 3D bioprinting technologies have been applied to the fabrication of a vessel-like structure embedded with cells. We can draw conclusions that: (a) both theory and the experiment proved that the material extrusion rate, the rotating shaft tangential velocity, and the platform movement speed have an obvious synchronous relationship, and (b) the cell survival rate was statistically analyzed, showing that the printed cell survival rate can reach 90%, which not only provides valid proof for the application of 3D bioprinting in the field of blood vessels, but also promotes the development of tissue engineering and regenerative medicine.
